# Current applications of multiparameter flow cytometry in plasma cell disorders

**DOI:** 10.1038/bcj.2017.90

**Published:** 2017-10-20

**Authors:** T Jelinek, R Bezdekova, M Zatopkova, L Burgos, M Simicek, T Sevcikova, B Paiva, R Hajek

**Affiliations:** 1 Department of Haematooncology, University Hospital Ostrava and Faculty of Medicine, University of Ostrava, Ostrava, Czech Republic; 2 Faculty of Science, University of Ostrava, Ostrava, Czech Republic; 3 Clinica Universidad de Navarra, Centro de Investigacion Medica Aplicada (CIMA), IDISNA, Pamplona, Spain; 4 Department of Clinical Haematology, University Hospital Brno, Brno, Czech Republic

## Abstract

Multiparameter flow cytometry (MFC) has become standard in the management of patients with plasma cell (PC) dyscrasias, and could be considered mandatory in specific areas of routine clinical practice. It plays a significant role during the differential diagnostic work-up because of its fast and conclusive readout of PC clonality, and simultaneously provides prognostic information in most monoclonal gammopathies. Recent advances in the treatment and outcomes of multiple myeloma led to the implementation of new response criteria, including minimal residual disease (MRD) status as one of the most relevant clinical endpoints with the potential to act as surrogate for survival. Recent technical progress led to the development of next-generation flow (NGF) cytometry that represents a validated, highly sensitive, cost-effective and widely available technique for standardized MRD evaluation, which also could be used for the detection of circulating tumor cells. Here we review current applications of MFC and NGF in most PC disorders including the less frequent solitary plasmocytoma, light-chain amyloidosis or Waldenström macroglobulinemia.

## Introduction

Plasma cell (PC) dyscrasias are a heterogeneous group of blood disorders characterized by the detection of a monoclonal paraprotein in serum or urine, which is often associated with the presence of clonal PCs in the bone marrow (BM) and eventually other tissues.^1^ With an estimated incidence of 6 cases per 100 000 persons per year, multiple myeloma (MM) represents the second most common hematologic malignancy and ~1% of all malignant tumors.^2^ MM is characterized by the presence of >10% of clonal BM PCs (based on morphological assessment) or biopsy-proven plasmocytoma, together with at least one of the so called ‘myeloma defining events’.^3^ Myeloma is virtually always preceded by an asymptomatic premalignant stage termed monoclonal gammopathy of undetermined significance (MGUS) that is present in about 3–4% of normal individuals over the age of 50 years.^4, 5^ The risk of progression of MGUS to MM or related disorders is about 1% per year.^6, 7^ Smoldering multiple myeloma (SMM) represents an intermediate clinical stage between MGUS and MM in which the risk of progression to malignant disease in the first 5 years after diagnosis is much higher, about 10% per year.^3, 8^ Amongst other, less frequent, PC dyscrasias there are: (i) plasma cell leukemia (PCL), (ii) AL amyloidosis (AL), (iii) Waldenström macroglobulinemia (WM), (iv) POEMS syndrome and (v) solitary plasmocytoma with or without minimal BM involvement (Table 1).

**Figure 1:**
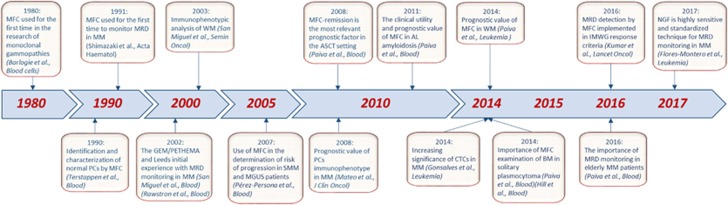
Time axis highlighting the most important discoveries concerning multiparameter flow cytometry and its use in plasma cell dyscrasias.

Multiparameter flow cytometry (MFC) immunophenotyping has been a mainstay in the diagnosis and monitoring of most hematologic malignancies.^9, 10, 11, 12, 13^ Together, with the patient’s clinical history, analytic results and morphological assessment of blood smears, MFC is also part of the initial diagnostic work-up, mainly because of its capacity to typically provide conclusive results within a few hours. As the importance of MFC has progressively increased in PC dyscrasias (Figure 1), its utility will be thoroughly reviewed in this manuscript.

## Detection of normal and pathological plasma cells

### Identification and enumeration of the PC compartment

The first step during the analysis of patients with PC dyscrasias at diagnosis and during follow-up is represented by the identification and enumeration of the PC compartment. PCs are end-stage antibody producing B-cells that are derived from antigen-activated B-cells generated in secondary lymphoid tissues. Early-stage PCs (generally called plasmablasts) can be found in peripheral blood (PB) during their recirculation from the tissues of origin seeking for survival niches (for example, in BM) where they evolve to long-living PCs. Plasmablasts lose CD20, express CD19, CD38^high^, CD45 and approximately half of them show reactivity for CD138.^14^ Although CD38 is a very promiscuous antigen ubiquitously expressed on all immune cells, its intensity is uniquely high on PCs,^15^ making it a reliable marker for PC gating. Conversely, CD138 (Syndecan-1) is specific to PCs (within hematopoietic cells) and, accordingly, has been found very useful in their identification. These two markers together with CD45, sideward (SSC) and forward (FSC) light scatter are recommended for accurate identification and enumeration of PCs.^16, 17^

### Immunophenotypic discrimination between normal vs pathologic plasma cells

It should be noted that no single phenotypic marker is sufficient to distinguish between normal/reactive plasma cells vs tumor plasma cells. Most BM normal PCs do not express pan B-cell markers such as CD20 or CD22, lack surface membrane immunoglobulins (smIg) and show polyclonal cytoplasmatic staining of light chains (cyKappa, cyLambda). Moreover, normal PCs show heterogeneous expression of CD19, CD27, CD45 and CD81. Thus, among normal BM PCs there are several phenotypically distinct subpopulations that display maturation-associated features according to the most commonly used markers CD19, CD27, CD45, CD56 and CD81. The majority of normal BM PCs are CD19^+^, CD45^dim^, CD56^−^ and CD81^+^, but >30% of them are CD19^−^, CD45^+^, CD56^+^ or CD81^−^, in multiple possible combinations.^17, 18^

There is compelling evidence that tumor PCs display different phenotypic features as compared to their normal counterparts. It was reported in 1998 a correlation between the presence of abnormal phenotypes detectable by flow cytometry, and the presence of tumor, clonal PCs; accordingly, fluorescent-activated cell sorting (FACS) of PCs with aberrant vs normal phenotypes (for example, bright expression of CD56) correlated with clonal vs polyclonal PCR V–D–JH products.^19^ Additional studies confirmed that tumor PCs typically show: (i) underexpression of CD19, CD27, CD38, CD45 and CD81, (ii) overexpression of CD28, CD33, CD56, CD117 and CD200 and (iii) asynchronous expression of CD20 and SmIg.^17, 20, 21, 22^ Except for CD117 that is almost never expressed on normal PCs, most of the expression patterns defined above can be found, individually (that is, not simultaneously), in small subsets of nPCs. This has become particularly evident with the advent of digital flow cytometers allowing for multidimensional combinations together with faster acquisitions and the measurement of higher number of cells. For example, it has been recently suggested that long-lived PCs downregulated CD19, CD38, CD45, CD81 and upregulated CD28 and CD56, which are some of the phenotypic hallmarks of tumor PCs. In fact, it could be speculated that such long-lived PCs could represent the normal cellular counterpart of many MM patients. Thus, it is currently recommended that the clonal nature of tumor PCs as defined by MFC, should be confirmed by the presence of simultaneous and multiple aberrant phenotypes together^23^ defined PC subsets with such aberrant phenotypes. Frequencies of these abnormal patterns of expression are summarized in Table 2.

### Practical considerations

It is well-accepted that the percentage of PCs is usually under-represented by MFC as compared to other cytologic methods. While there are several factors that could be responsible for this phenomenon, the most probable explanation is that tumor PCs are associated with lipid enriched BM spicules in the morphology slides, as opposed to lipid-depleted liquid BM analyzed by MFC.^24^ That notwithstanding, higher PB hemodilution has typically characterized BM samples devoted to MFC (and other laboratory tests), as the first BM pull has been dedicated to morphology slides. Highly representative and non-diluted BM samples are crucial for valid and precise results particularly during MRD evaluation, and it is highly recommendable to use the first BM pull for flow-based MRD assessment. Another factor resulting in the underestimation of BM PCs is the loss of PCs during sample preparation due to the potentially higher susceptibility of PCs for mechanical damage.^22^ Accordingly, we recommend the adoption of methods that have been extensively validated and demonstrated superior PC recovery as compared to other methods.^23^ It should be noted though that most current applications of MFC take place in disease stages or disease entities in which PCs are <1% of total nucleated BM cells and in which a mixture of normal and tumor PCs coexist, making it the only cytologic technique with enough sensitivity for accurate quantification and characterization of tumor PCs.

## The transition from MGUS to smoldering and active MM

### Role of MFC in differential diagnosis

From a clinical point of view, one of the most evident roles of routine MFC is during patients’ differential diagnostic work-up. First, to distinguish between prominent but reactive and benign plasmocytosis vs clonal and potentially malignant PC dyscrasias.^25^ Second, to recognize B-cell non-Hodgkin lymphomas (B-NHL) with extensive plasmacytic differentiation such as lymphoplasmacytic lymphoma (LPL, WM) or marginal zone lymphoma (MZL). The distinction can be made by careful identification of small B-cell clones that may be below the limit of detection of morphology or immunohistochemistry (IHC), together with comprehensive evaluation of the phenotypic profile of tumor PCs.^26^ Third, to confirm the diagnosis of rare IgM myeloma cases based on distinct PC phenotype from other IgM producing B-cell disorders.^25^ Fourth, to help discriminate between MGUS vs SMM vs active MM based on the percentage of nPCs within the bone marrow PC (BMPC) compartment. Twenty years have passed since Ocqueteau *et al.* described that MGUS is characterized by the co-existence of normal PCs and tumor PCs (100% of cases) whereas in MM this finding is less frequent (22% of cases). Moreover, only 1.5% of MM patients had more than 3% of nPCs, whereas 98% of MGUS patients had more than 3% of nPCs. Therefore, a proposed cut-off of >5% of residual normal PCs (within the BMPC compartment) has been found to help in the discrimination between MGUS and active MM.^19, 27, 28^ That notwithstanding, one of the most useful applications of this threshold has been found in SMM, in which it would allow to discriminate patients with MGUS-like vs MM-like phenotypic profiles and different risk of progression.^29, 30^

### Role of MFC in providing prognostic information

Prognostic information provided by MFC-based evaluation of the BM PC compartment is not that widely used as other prognostic factors such as International staging system (ISS), cytogenetic abnormalities or lactate dehydrogenase.^31, 32, 33^ Nevertheless, there is growing evidence suggesting that MFC can be useful to: (i) predict the risk of transformation of MGUS and SMM into active MM; (ii) identify a small subgroup of symptomatic MM patients with an exceptionally favorable prognosis (that is, those with MGUS-like profile); and (iii) offer prognostic information based on the immunophenotype of tumor PCs. The prognostic value of circulating PCs (CTCs) will be reviewed in a separate chapter.

The model predicting the risk of transformation of MGUS and SMM into symptomatic MM was designed by the Spanish group and is based on the presence of >95% of tumor PCs within the BM PC compartment. Thus, MGUS patients who fulfill this criterion had a cumulative probability of progression into symptomatic MM at 5 years of 25 vs 5% of those who had <95% of tumor PCs. Similarly, using the same criterion in SMM patients, a cumulative probability of progression into active MM at 5 years was 64% vs only 8%.^29, 34^ There are ongoing efforts to standardize MFC and develop automated models to predict risk of transformation in SMM.^35^ Conversely, a subgroup of newly diagnosed MM (NDMM) patients who have, at the time of diagnosis, more than 5% of residual normal PCs from all BM PCs, display unique clinical and biological characteristics such as lower BM infiltration by PCs, higher hemoglobin levels, lower frequency of immune paresis and others. Paiva *et al.*^36^ have shown in a cohort of 594 uniformly treated NDMM patients, that this subgroup (14% of analyzed patients) had significantly longer progression-free survival (median PFS, 54 vs 42 months, *P*=.001) and overall survival (median OS, not reached vs 89 months, *P*=0.04) than patients with ⩽5% of normal PCs in the BM PC compartment. Driven by these observations, the Spanish Myeloma Group subsequently developed an automated flow cytometric algorithm that recognizes, amongst NDMM patients, those with the so-called MGUS-like phenotype that display a highly favorable prognosis. According to this algorithm, 8% of NDMM patients were identified with an MGUS-like profile. MGUS-like cases had unprecedented longer time-to-progression (TTP) and OS (∼60% at 10 years; *P*<0.001) rates. Importantly, MGUS-like MM patients failing to achieve complete remission (CR) showed similar TTP (*P*=0.81) and OS (*P*=0.24) vs cases attaining CR. In fact, identifying these patients could be clinically relevant because they may experience favorable outcomes (may not progress despite evidence of disease, as MGUS patients do) in the absence of CR. This group of patients may represent an exception to the rule: ‘the deeper the response, the longer the survival’ and should not be over-treated in pursuit of reaching deep responses.^30^ That notwithstanding, MGUS-like patients reaching MRD-negativity after first-line treatment have the best outcomes of all MM patients (median PFS of 12 years and a 10-year OS rate of 94%).^37^

Many studies have been conducted to evaluate the prognostic significance of the immunophenotype of myeloma PCs, but only a few antigens have shown to be of prognostic value. CD117 (proto-oncogene c-KIT) is a receptor tyrosine kinase normally expressed by mast cells and hematopoietic progenitors in the BM, but absent during B-cell maturation from early precursors to PCs.^38^ This antigen is aberrantly over-expressed in ~70% of MGUS patients and 30% of MM patients. Although its expression is related to oncogenic transformation in other malignancies, in MM it is associated with favorable outcomes. This occurs hypothetically because of the altered homing of clonal CD117+ PCs that are redirected towards the neutrophil precursor niches, thus giving space for maintenance of residual nPCs.^39, 40^ Conversely, adverse prognosis is associated with expression of CD28 (T-cell co-stimulatory receptor) that is positive in approximately 35% of MM cases. This adverse effect was originally attributed to a strong association with high-risk cytogenetic abnormalities, but recently an alternative explanation has been proposed based on the pro-survival effect of plasma cell–dendritic cell interactions.^39, 41^ According to Mateo *et al.*, three separate risk groups can be identified based on the cohort of 685 uniformly treated NDMM patients: poor (CD28+/CD117−), intermediate (CD28+/CD117+ or CD28−/CD117−) and good (CD28−/CD117+) with corresponding median OS of 45 months, 68 months and not reached, respectively. This study also revealed an adverse prognostic role of CD19 (part of the B-cell receptor, expressed only in B-cell lineage) that is positive only in 5% of MM patients.^39^ The antigen CD19 is regulated by CD81 that is a glycoprotein from the tetraspanin family expressed in 45% of MM cases. Paiva *et al.*^42^ described and validated the expression of CD81 as a negative prognostic marker for symptomatic MM patients as well as a marker for the risk of progression in SMM patients. Recently, a new maturation axis of normal PCs, based on these two antigens—CD19 and CD81, was proposed. Three BM PC subsets with progressively increased differentiation from CD19+/CD81+ into CD19−/CD81+ and CD19−/CD81−PCs were identified. Authors also demonstrated that MM cells fit into such a model of normal BM PC differentiation and revealed that 59% of 225 NDMM patients had fully differentiated (CD19−CD81−) clones, 38% intermediate-differentiated (CD19-CD81+), and 3% less-differentiated (CD19+CD81+) clones. The latter patients had a dismal outcome, and PC differentiation emerged as an independent prognostic marker for PFS and OS.^18^ The prognostic impact of other frequently used markers such as CD20, CD45, CD56 or CD200 is not strong enough to consider them to be independent prognostic markers.^22, 43^

The assessment of PC ploidy and proliferation have been long shown to provide prognostic information in MM.^44, 45^ However, it should be noted that while the detection of both non-hyperdiploid DNA content and ⩾1% PCs in S-phase are of independent prognostic value for OS in newly diagnosed MM patients, treatment with bortezomib-based regimens might abrogate the inferior OS of patients with ⩾1% PCs in S-phase.^46^ Thus, the prognostic value of MFC-based DNA studies should be revisited in the era of modern treatment strategies.

### Role of MFC in response assessment

Traditional response criteria in MM have been based on the evaluation of serum and urine monoclonal protein concentrations by electrophoresis or immunofixation as a surrogate for tumor burden.^47^ The original definition of complete response (CR) required <5% of PCs in the BM, irrespective of their clonal nature, together with negative immunofixation and disappearance of any soft tissue plasmocytomas.^48^ This definition was further refined to stringent complete response (sCR) by adding the normalization of the serum free light chain (sFLC) ratio and the absence of clonal PCs in BM assessed by IHC.^49^ Nowadays, there is a direct relationship between the depth of the response, particularly CR, and prolonged PFS and OS. This has been confirmed amongst NDMM transplant-eligible as well as elderly patients and also in relapsed/refractory patients.^50, 51, 52^ This concept of ‘the deeper the response, the longer the survival’ is valid for the vast majority of MM patients with the exception of specific molecular subgroups or those with an MGUS-like phenotypic profile.^30, 53^ The recent progress in effective treatment strategies for MM was translated in considerably better outcomes with practically 100% of patients responding to treatment and >50% reaching CR. These advances created an unmet need to implement highly sensitive techniques able to determine the presence of very low numbers of clonal PCs within the BM— that is, minimal residual disease (MRD) (Table 3). In the most recent International Myeloma Working Group (IMWG) consensus guidelines for response assessment, new MRD criteria were introduced and an MRD-negative status assessed by next-generation flow (NGF) cytometry was included.^52^

**Figure 2:**
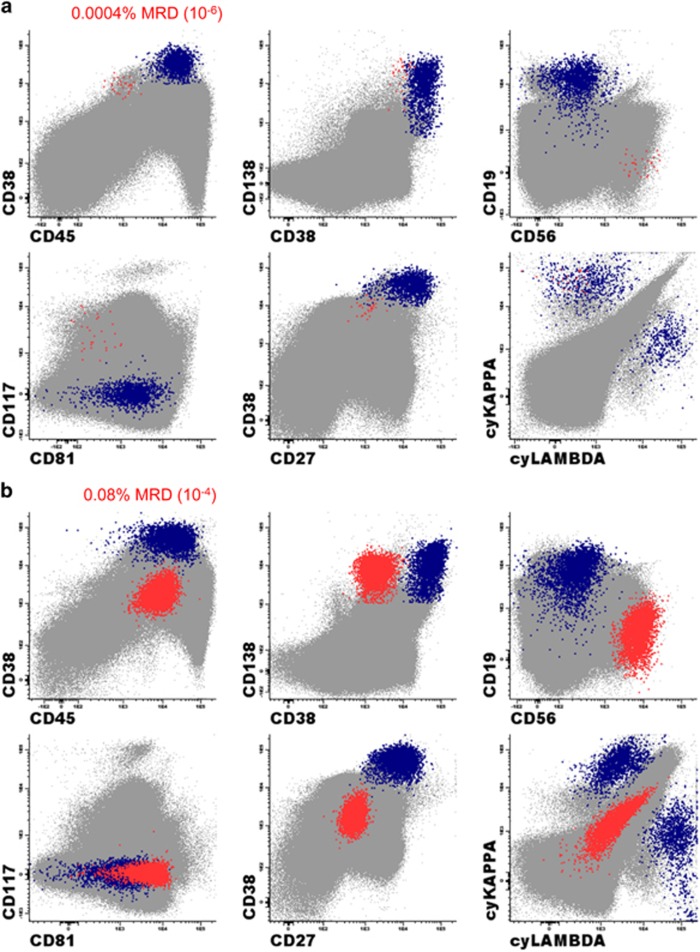
Example of MRD analysis in MM using next generation flow approach and Infinicyt software (Cytognos). (**a**) Bone marrow PC compartment represents 0.04% of total nucleated cells including 98.5% of normal PCs (blue) and 1.5% of aberrant PCs (red). These aberrant plasma cells represent 0.0004% of total nucleated cells translating in MRD positive result reaching the sensitivity of 10^−6^. The typical aberrant phenotype: CD45^−^/CD38^dim^/CD19^−^/CD56^+^/CD27^−^/CD81^−^/CD117^+^/cyKappa^+^. (**b**) NGF is optimal tool also for follow-up of patients with non-secretory multiple myeloma. Bone marrow PC compartment represents 0.16% of total nucleated cells including 50% of normal PCs (blue) and 50% of aberrant PCs (red). Aberrant PCs in this case have rare immunophenotype with CD38- and lack of cytoplasmic staining of kappa or lambda light chains: CD45^+^/CD38^−^/CD19^−^/CD56^+^/CD27^−^/CD81^+^/CD117^−^/cyKappa^−^/cyLambda^−^.

The concept of MFC-based monitoring of MRD and its prognostic value was introduced in 2002 by Spanish and UK groups.^54, 55^ Paiva *et al.* demonstrated in 295 uniformly treated NDMM patients receiving HDT/ASCT that MRD-negativity at day +100 after ASCT translated to significantly prolonged PFS (PFS; median 71 vs 37 months, *P*<0.001) and OS (OS; median not reached vs 89 months, *P*=0.002).^56^ Similarly, Rawstron *et al.* reported that in 397 patients from the UK MRC Myeloma IX trial, MRD negativity at day +100 after ASCT is highly predictive for favorable outcomes.^57^ Interestingly, the combined evaluation of baseline cytogenetics/FISH and flow MRD evaluation at day +100 after ASCT provides powerful risk stratification and identifies a subgroup of patients with dismal outcomes of OS of only 2 years (those with high-risk FISH and MRD positivity).^58^ These studies were performed using 4- and 6-color (the first generation) MRD methods reaching sensitivity of 10^−4^ (ability to identify 1 PC in 10 000 cells, that is 0.01%). On the basis of the second-generation flow MRD methods mostly defined by the usage of 8 colors and interrogation of a higher numbers of cells, Paiva *et al.* showed that the MRD status is one of the most important and independent prognostic factors also in elderly transplant-ineligible patients (*n*=162). The sensitivity of this method reached 10^−5^ (ability to identify 1 PC in 100 000 cells, that is, 0.001%). This study also showed that it is important to reach the sensitivity of 10^−5^ as the patients being MRD positive, even at very low levels below 10^−4^, had significantly worse outcomes.^59^ A highly sensitive and fully standardized approach called NGF for MRD detection in MM was implemented by the EuroFlow Consortium recently. Optimized two 8−color tube panels with an established bulk-lysis procedure allow the acquisition of ⩾10^7^ cells/sample reaching sensitivity close to 10^−6^ (with the limit of detection being 20 clonal PCs among 10^7^ evaluated BM cells, that is, 0.0002% and limit of quantification 50 clonal PCs among 10^7^ BM cells)^23^ Figure 2. Moreover, NGF MRD is applicable to virtually all patients (⩾ 98%) and incorporates a quality check of patient BM sample via simultaneous identification of a significant decrease in the non-PC BM population (such as CD117^+++^ mast cells, nucleated red cells, 117^+^ myeloid precursors or CD19^+^ CD45^lo^ CD38^+^ B-cell precursors). This information is crucial to recognize potentially hemodiluted BM aspirates that may lead to false-negative results.^23, 60^ The exact preparation procedures, staining, acquisition and reporting of flow MRD are described in detail in recently published guidelines.^16, 61^ Flow MRD represents a fast, highly sensitive, standardized, cost-effective and widely available technique for MRD evaluation in MM and is likely to soon be implemented in routine clinical practice as one of the most important clinical endpoints and sensitive readouts of treatment efficacy.

Other alternative to MFC-based method of MRD evaluation represent molecular techniques, mainly ASO-PCR (allele-specific oligonucleotide PCR) and NGS (next-generation sequencing). Both methods reach high levels of sensitivity (down to 10^−5^–10^−6^) and do not require immediate sample processing in contrary to MFC method (sample should be processed within 36–48 h after BM harvest). On the other hand, these methods have some disadvantages: (i) lower applicability (ASO-PCR~60–70%, NGS~90%), (ii) requirement of diagnostic sample to identify patient-specific clonotypic sequences, (iii) higher financial costs and (iv) methodologically complex and laborious methods difficult to implement in routine clinical practice.^62, 63, 64^

## Circulating plasma cells

Long-living BM PCs do not circulate under physiological conditions in PB, but in patients with certain monoclonal gammopathies (MG) tumor cells can egress from BM into PB as circulating tumor cells (CTCs). Accordingly, the presence of CTCs was documented not only in symptomatic MM, but also in SMM and MGUS.^65, 66^ Paiva *et al.*^67^ demonstrated that in MM the number of CTCs fluctuates throughout the day, following a circadian rhythm similar to CD34^+^ cells, suggesting that CTCs may egress to PB to colonize other sites during the patients’ resting period.

**Figure 3:**
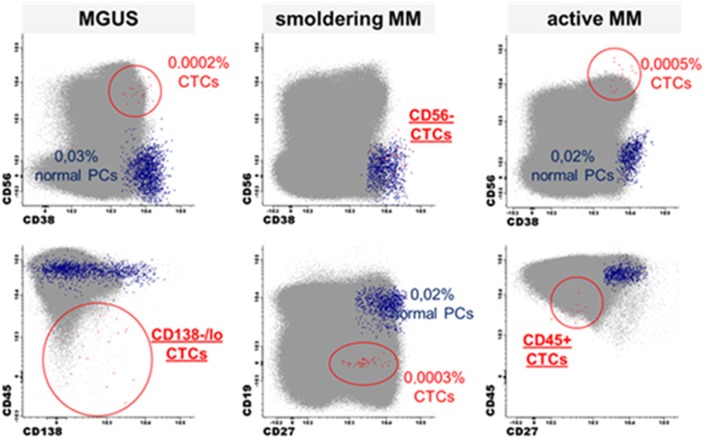
Next-generation flow approach used for identification of CTCs in MGUS, smoldering MM and active MM patients.

The detection of CTCs in NDMM patients by conventional morphology is low (20% of all cases).^68^ By using more sensitive MFC (Figure 3), CTCs are detected in approximately 70–87% of all NDMM patients^65, 69, 70^ and up to 60% of MGUS patients.^71^ The number of CTCs is an independent prognostic factor in NDMM patients as well as in AL amyloidosis, and their presence is associated with shorter survival.^66, 72, 73, 74^ CTCs are associated with an increased risk of malignant transformation in MGUS^72^ and SMM^75, 76^ and they are also a negative prognostic factor in RRMM.^77^ It is expected that particularly in SMM, MFC could become a convenient method (that is, non-invasive) to identify patients with high-risk of progression to MM before they develop end-organ damage.^76^ CTC FACS could also be used as a minimally-invasive method to interrogate patients’ genomic landscape over time.^78^

From a biological standpoint, in comparison to BM PCs, CTCs show down-regulation of some surface markers such as several integrins (CD11a/CD11c/CD29/CD49d/CD49e), adhesion molecules (CD33/CD56/CD117/CD138), and activation molecules (CD28/CD38/CD81).^67^ However, the cause and mechanism of PC egression from BM remains poorly understood, as one of the hallmarks of aPCs in early stages of the disease is their dependence on the BM microenvironment. Possible reasons for their migration/expansion may include changes in angiogenesis and the subsequent increase in their proliferative rate, higher incidences of genetic abnormalities or changes in the expression profile of adhesion molecules.^67, 79, 80^ An important question remains unanswered: is the presence of CTCs linked to the natural development of the disease or does it identify a separate biological subgroup of patients?^80, 81, 82^ It was shown that CTCs are mostly quiescent, but they could have a higher clonogenic potential to their paired BM counterparts. This fact could explain their potential ability of dissemination. In such cases, CTCs would represent a unique subpopulation of BM clonal PCs.^67^ Recently, Bretones *et al.* compared exomes of FASC sorted BM PCs, CTCs and PCs from extramedullary (EM) tissues in 6 MM patients with EM disease and demonstrated the presence of systematic inter-tissue heterogeneity (though not for targetable mutations). CTCs displayed the highest frequency of shared mutations with the two other clones and the lowest number of private mutations, suggesting that while CTCs may bridge BM and EM myeloma, there is continuous genomic evolution once different clones have seeded in their respective niches.^83^

## Solitary plasmocytoma

Solitary plasmocytomas (SPs) are rare PC dyscrasias (<5%) that are characterized by the presence of bone (solitary bone plasmocytoma—SBP) or extramedullary soft tissue (extramedullary plasmocytoma—EMP) infiltrates of tumor PCs in the absence of any clinical, laboratory and radiologic features of MM.^84, 85^ Local radiotherapy, with or without surgical excision, is the recommended treatment of choice for these patients, and achieves high OS rates. Patients with SBP may progress to MM at a rate of approximately 40–50%, with lower rates of progression for EMP.^85, 86^ According to the 2014 IMWG criteria, SPs were divided into 2 entities: with or without minimal BM involvement based on the evaluation of the presence of tumor PCs by MFC, Table 1. In fact, the presence of tumor PCs in patients with SP has become the most important prognostic marker defining the risk of progression. Paiva *et al.* have detected tumor PCs in 49% (17/35) of SBP patients and in 38% (11/29) of EMP patients. Seventy-one percent of flow-positive vs only 8% of flow-negative SBP patients evolved to MM (hazard ratio, 17.4; *P*<0.001). No significant differences were observed among EMP cases. Almost identical results were published by a UK group^84^ enhancing the importance of MFC for the sub-classification of patients with SP and leading to its new classification as noted above.

## AL amyloidosis

Immunoglobulin light-chain amyloidosis (AL amyloidosis) is a rare PCD with an incidence of 10 patients per million cases per year but represents the most common of systemic amyloidoses.^87^ This often fatal disorder is characterized by the presence of a usually small indolent clone of BM PCs that produce misfolded monoclonal light chains of κ or most predominantly λ isotype formatting insoluble fibrils causing damage to vital organs, Table 1.^88^ In AL, MFC can be useful to confirm the presence of underlying tumor PCs responsible for the deposition of the amyloid light chains in tissue biopsies. This underlying tumor PC clone is present and revealed by MFC in virtually all AL patients.^89, 90^ There are only few MFC studies focusing on its prognostic value due to the rarity of this disease. Quantification of the BM PC compartment by MFC was found to be a significant prognostic factor for OS (<1 vs >1% BMPC cutoff; 2-year OS rates of 90 vs 44%, *P=*0.02) in the cohort of 35 newly diagnosed AL patients. Moreover, detecting persistent nPCs at diagnosis identified a subgroup of patients with prolonged OS (cut off >5 vs <5% of nPCs/BMPC, 2−year rates of 88 vs 37%, *P*=0.01). In these series, 49% (17/35) of AL patients had >5% of nPCs/BMPC at diagnosis.^91^ More recently, a very similar study was published by the Mayo Clinic group (*n*=173) confirming some of the above mentioned results using a different cutoff for quantification of BM PCs (2.5%).^92^

## Waldenström macroglobulinemia

Waldenström macroglobulinemia (WM) was originally described by Jan Gösta Waldenström in 1944.^93, 94^ This disease is defined as a lymphoplasmacytic lymphoma associated with the monoclonal immunoglobulin IgM and BM infiltration by small IgM-producing clonal B-lymphocytes that may exhibit PC differentiation.^95^ An incidence of 4 cases per million persons per year ranks this disease amongst rare disorders.^93^ The clinical presentation of patients with WM can be highly variable as the signs and symptoms are not only due to the infiltration of BM or lymphoid organs (anemia, hepatosplenomegaly and so on), but also due to the specific physiochemical and immunological properties of monoclonal IgM (peripheral neuropathy, hyperviscosity and so on).^96^ Similarly to MM, it is supposed that virtually all cases of WM have gone through the benign stages of IgM MGUS and smoldering WM (SWM) before developing clinical symptoms. The hallmark of these three entities is the presence of the monoclonal IgM protein. The difference between IgM MGUS and smoldering WM is in the BM infiltration by B-lymphocytes (with a cutoff of 10% defined by morphology). Smoldering WM, in contrast to symptomatic WM, lacks any disease-associated clinical symptoms, thus not requiring any treatment.^95, 97^ Advances in understanding the molecular pathogenesis of WM have been paved by the discovery of a recurrent somatic mutation in MYD88^L265P^ that is present in more than 90% of symptomatic WM patients, but also in at least half of IgM MGUS patients suggesting its role as an early oncogenic event.^98, 99^ The most plausible normal counterpart of the WM clonal B-cell seems to be a CD22^low+^/CD25^+^ memory B-cell as the cell of tumor origin,^97^ even though this question has still not been fully answered.^100^

MFC plays a significant role in the differential diagnostic work-up. It is particularly useful to distinguish WM from other B-cell lymphoproliferative disorders as well as from related IgM monoclonal gammopathies (that is, IgM MGUS, IgM myeloma) based on the specific immunophenotype of WM B-cells and PCs.^96^ The co-existence of a clonal B-cell population and the same light-chain-isotype clonal plasma cell population within the BM compartment is usually accompanied by an increased number of mast cells, thus representing typical findings.^101^ The most common immunophenotype of WM clonal B-cells can be described as follows: CD19^+^/CD22^low+^/CD23^−^/CD25^+^/CD27^+^/SmIgM^+^. In more detail, clonal B-cells systematically show SmIgM^+^ expression, with homogenously expressed CD22^low+^ (81% of cases) and CD25^+^ (89% of cases). CD79b and CD81 are positive in all cases, whereas a heterogeneous bimodal pattern of expression can be observed for CD27 (51% of cases), CD38 (50%) and CD200 (62%). CD305 (LAIR1) is not expressed in up to 69% of cases in contrast to its bimodal heterogeneous expression on normal B-cells. The absence of the expression of CD5, CD10, CD11c and CD103 (in 95%, 100 96 and 100% of cases, respectively) is in contrast with most other mature lymphoid malignancies (chronic lymphocytic leukemia, mantle cell lymphoma, follicular lymphoma and hairy cell leukemia) except of marginal zone lymphoma (MZL), thus helping in their distinction. The most useful markers to discriminate between WM and MZL, that possess the overlapping phenotype in some cases, were SmIgM and CD79b, both over-expressed in WM, and CD305 upregulated in MZL.^97, 102^

The antigenic profile of clonal PCs in WM resembles more that of normal PCs and clearly differs from that of MM patients (for example, CD19^−^/CD27^−^/CD45^−^ and/or CD56^+^). Bone marrow PCs from IgM MGUS to symptomatic WM patients show a progressively increased frequency of CD19^+^/CD20^+^/CD45^+^ and SmIgM^+^ cells, together with a complete loss of CD56 reactivity. This suggests that the PC compartment is enriched in more immature clonal PCs with the plasmablastic phenotype.^103^ The different antigenic profile of clonal PCs can have a valuable input to distinguish WM from IgM MM, together with the MYD88 mutation status (not present in IgM MM) and the presence of t(11; 14) (does not occur in WM but with a high incidence in IgM MM).^93, 104, 105^

Flow cytometry may be a useful tool in the discrimination between IgM-related disorders. The thresholds of BM B-cell infiltration (>10%) and the degree of B-cell clonality (100%) are highly specific to exclude the diagnosis of IgM-MGUS, but not otherwise to identify WM patients. However, flow cytometry can also serve as a valuable prognostic tool in patients with smoldering and symptomatic WM. Patients with smoldering WM who have more than 10% of BM B-cell infiltration and who show full light-chain restriction of the B-cell compartment are at a higher risk of progression to symptomatic disease (median TTP of 26 vs 145 months, *P*⩽0.001). Similarly, patients with symptomatic WM and with 100% of light chain restricted B-cells show inferior survival vs those who maintain some polyclonal B-cells (median OS of 44 vs 78 months, *P*=0.001). To note, full light chain restriction of the PC compartment has no effect on both TTP and OS in smoldering and symptomatic WM patients, respectively.^102^

Finally, the role of MFC was also investigated during the response assessment. García-Sanz *et al.*^106^ described in 42 WM patients that post-treatment BM residual disease status >5% of monoclonal B-cells was highly predictive of short PFS and OS, independently on hematological response. With the availability of more effective therapies for WM, it will be critical to combine sensitive and comprehensive monitoring of clonality in both B-cells and PCs together with the evaluation of serum M-protein, since the complete eradication of only clonal B-cells but not PCs (for example, through anti-CD20 immunochemotherapy) may translate into a favorable outcome despite sub-optimal response according to current criteria (that is, persistent M-protein).

## Conclusion

MFC immunophenotyping has become routinely used in the management of patients with PC dyscrasias. MFC plays a significant role during the differential diagnostic work-up, when this technique can provide relatively fast and conclusive results, thus helping to distinguish between malignant and reactive conditions as well as classifying different monoclonal gammopathies and other lymphoproliferative disorders. Flow cytometry can also be useful in predicting outcomes not only in MGUS/SMM/MM, but also in patients with SWM/WM, AL and SP. The obtained information could be particularly beneficial in maintaining a closer surveillance of patients at a higher risk of progression and in identifying the subgroup of patients with exceptionally favorable outcomes. Flow-based MRD monitoring in MM, particularly after the implementation of NGF, represents a fast, highly sensitive, standardized, cost-effective and widely available technique. The importance of defining the MRD status is indisputable in MM patients and flow MRD is progressively being implemented in routine clinical practice as one of the most relevant treatment end-points. Immunophenotyping has greatly contributed also in the research of monoclonal gammopathies and may be of significant importance in the upcoming era of immunotherapy, especially in defining and monitoring therapeutic targets such as CD38, SLAMF7, PD-L1, BCMA and many others that are yet to be discovered.^107, 108^

## Figures and Tables

**Table 1:** Definitions of plasma cell related disorders (adopted from Rajkumar *et al.*,^3^)

*Title*	*Definition*
MGUS	Serum monoclonal protein (non-IgM type) <30 g/l
	Clonal bone marrow plasma cells <10%
	Absence of end-organ damage such as hypercalcaemia, renal insufficiency, anaemia, and bone lesions (CRAB) or amyloidosis that can be attributed to the plasma cell proliferative disorder
SMM	Both criteria must be met:
	• Serum monoclonal protein (IgG or IgA) ⩾30 g/l or urinary monoclonal protein ⩾500 mg per 24 h and/or clonal bone marrow plasma cells 10–60%
	• Absence of myeloma defining events or amyloidosis
MM	Clonal bone marrow plasma cells ⩾10% or biopsy-proven bony or extramedullary plasmacytoma
	Evidence of any of myeloma defining events
PCL	Presence of >20% of clonal plasma cells in peripheral blood and/or the absolute number of circulating plasma cells exceeding 2 × 10^9^/l in peripheral blood
Solitary	Biopsy-proven solitary lesion of bone or soft tissue with evidence of clonal plasma cells
Plasmacytoma	Normal bone marrow with no evidence of clonal plasma cells
	Normal skeletal survey and MRI (or CT) of spine and pelvis (except for the primary solitary lesion)
	Absence of end-organ damage such as hypercalcaemia, renal insufficiency, anaemia, or bone lesions (CRAB) that can be attributed to a lymphoplasma cell proliferative disorder
Light-chain	Abnormal FLC ratio (<0·26 or >1·65)
MGUS	Increased level of the appropriate involved light chain (increased κ FLC in patients with ratio >1·65 and increased λ FLC in patients with ratio <0·26)
	No immunoglobulin heavy chain expression on immunofixation
	Absence of end-organ damage such as hypercalcaemia, renal insufficiency, anaemia, and bone lesions (CRAB) or amyloidosis that can be attributed to the plasma cell proliferative disorder
	Clonal bone marrow plasma cells <10%
	Urinary monoclonal protein <500 mg/24 h
AL	Presence of an amyloid-related systemic syndrome (eg, renal, liver, heart, gastrointestinal tract, or peripheral nerve involvement)
	Positive amyloid staining by Congo red in any tissue (eg, fat aspirate, bone marrow, or organ biopsy)
	Evidence that amyloid is light-chain-related established by direct examination of the amyloid using mass spectrometry-based proteomic analysis, or immunoelectronmicroscopy
	Evidence of a monoclonal plasma cell proliferative disorder (serum or urine monoclonal protein, abnormal free light-chain ratio, or clonal plasma cells in the bone marrow)
IgM-MGUS	Serum IgM monoclonal protein <30 g/l
	Bone marrow lymphoplasmacytic infiltration <10%
	No evidence of anemia, constitutional symptoms, hyperviscosity, lymphadenopathy, hepatosplenomegaly or other end-organ damage that can be attributed to the underlying lymphoproliferative disorder
Smoldering WM	Presence of serum IgM monoclonal protein
	Bone marrow lymphoplasmacytic infiltration >10%
	No evidence of anaemia, constitutional symptoms, hyperviscosity, lymphadenopathy, hepatosplenomegaly, or other end-organ damage that can be attributed to the underlying lymphoproliferative disorder
WM	Presence of serum IgM monoclonal protein
	Bone marrow lymphoplasmacytic infiltration >10%
	Evidence of anaemia, constitutional symptoms, hyperviscosity, lymphadenopathy, hepatosplenomegaly, or other end-organ damage that can be attributed to the underlying lymphoproliferative disorder
POEMS	Polyneuropathy
Syndrome	Monoclonal plasma cell proliferative disorder (almost always λ)
	Any one of the following three other major criteria:
	• Sclerotic bone lesions
	• Castleman’s disease
	• Elevated levels of VEGFA
	Any one of the following six minor criteria:
	• Organomegaly (splenomegaly, hepatomegaly, or lymphadenopathy)
	• Extravascular volume overload (oedema, pleural eff usion, or ascites)
	• Endocrinopathy (adrenal, thyroid, pituitary, gonadal, parathyroid, pancreatic)
	• Skin changes (hyperpigmentation, hypertrichosis, glomeruloid haemangiomata, plethora, acrocyanosis, flushing, white nails)
	• Papilloedema
	• Thrombocytosis/polycythaemia

**Table 2:** List of the most relevant antigens for the detection of aberrant plasma cells in multiple myeloma

*Antigen*	*Normal plasma cell immunophenotype*	*Aberrant plasma cell immunophenotype*	*Percentage of patients with aberrant phenotype*	*Reference*
CD19	+	−	95%	^30^
				^18^
CD20	−	Dim +	17–30%	^30^
				^22^
CD27	++	− or dim +	40–68%	^22^
CD28	−/weak	+	15–45%	^48^
				^109^
CD33	−	+	15–18%	^110^
				^111^
CD38	++	Dim +	92%	^62^
				^26^
CD45	+	−	72–73%	^62^
				^112^
CD54	+	Dim +	60–80%	^113^
				^114^
CD56	−	++	60–76%	^115^
				^116^
				^117^
CD81	+	− or dim +	45%	^42^
				^18^
CD117	−	+	30–37%	^37^
				^30^
CD200	weak	+/++	65–86%	^20^
				^118^
SmIg	−	+	30%	^26^
CD319 (SLAMF7, CS1)	+	+	90–97%	^119^
				^120^
BCMA	+	+	100%	^108^

**Table 3:** Results of the most relevant studies using multiparameter flow cytometry for detection of minimal residual disease in multiple myeloma

*Setting*	*Method*	*LOD*	*Number of patients*	*CR (%)*	*MRD-negativity (%)*	*PFS (MRD-* vs *MRD+)*	P-*value*	*OS (MRD-* vs *MRD+)*	P*-value*	*Reference*
CT or ASCT	4-color MFC	10^−4^	87	39/87 (45%)	23/87 (26%)	60 m vs 34 m	0.02	NA	^−^	^48^
ASCT	3-color MFC	10^−3^–10^−4^	45	33/45 (73%)	24/45 (56%)	35 m vs 20 m	0.03	76 vs 64% at 5-years	0.28	^49^
ASCT	4-color MFC	10^−4^	295	147/295 (50%)	125/295 (42%)	71 m vs 37 m	<0.001	NR vs 89 m	0.002	^50^
Elderly	4-color MFC	10^−4^–10^−5^	102	44/102 (43%)	24/102 (24%)	90 vs 35% at 3-years	<0.001	94 vs 70% at 3-years	0.08	^90^
ASCT	4-color MFC	10^−4^–10^−5^	241, CR	241 (100%)	154/241 (64%)	86 vs 58% at 3-years	<0.001	94 vs 80% at 3-years	0.001	^52^
ASCT	6-color MFC	10^−4^	397	214/394 (54%)	246/394 (62%)	29 m vs 14 m	<0.001	81 m vs 59 m	0.02	^51^
ASCT	7-color MFC	10^−5^	31	18/31 (58%)	21/31 (68%)	100 vs 30% at 3-years	NA	NA	^−^	^121^
R/R	4-color MFC	10^−4^	52, CR	52 (100%)	24/52 (46%)	75 m vs 14 m	0.03	NA	^−^	^122^
Elderly	4 & 8-color	10^−5^	162	81/162 (50%)	54/162 (34%)	Median TTP: MRD-ve: NR CR & MRD+ve: 20 m <CR & MRD+ve: 11 m	<0.001	3 year OS: MRD-ve: 67% CR & MRD+ve: 53% <CR & MRD+ve: 60%	0.19	^53^
NA	4 & 6-color	10^−4^	78, CR	78 (100%)	34/78 (44%)	29.2 m vs 13.8 m	0.009	110.7 m vs NR	0.94	^123^
Follow-up	NGF	10^−5^	110 ⩾VGPR	71/110 (64%)	convent. flow: 37/110 (34%) NGF: 52/110 (47%)	75% NR vs 10 m	0.01	NA	^−^	^54^
RVD+SCT										
RVD	7-color MFC	10^−4^	350 350	205/350 (59%) 169/350 (48%)	220/278 (79%)^a^, 171/265 (65%)^a^	adjusted HR=0.30	*P*<0.001	adjusted HR=0.34	*P*<0.001	^124^

Abbreviations: ASCT autologous stem cell transplantation; CR, complete remission; CT, Chemotherapy; HR, hazard ratio; m, month; MFC, multiparameter flow cytometry; MRD, minimal residual disease; NA, data not available; NGF, next-generation flow; OS, overall survival; PFS, progression-free survival; R/R, relapse/refractory; RVD, lenalidomide, bortezomib and dexamethasone; SCT, stem cell transplantation; VGPR, very good partial response.

a MRD evaluated in patients reaching CR or VGPR.
