# Current applications of multiparameter flow cytometry in plasma cell disorders

**DOI:** 10.1038/bcj.2017.101

**Published:** 2018-01-19

**Authors:** T Jelinek, R Bezdekova, M Zatopkova, L Burgos, M Simicek, T Sevcikova, B Paiva, R Hajek

**Correction to:** *Blood Cancer Journal* (2017) **7**, e617; doi:10.1038/bcj.2017.90; published online 20 October 2017

Since the publication of this paper the authors have noticed that some of the references cited in the text were not numbered correctly. A duplicate reference in the reference list was removed during the production process but the references were not correctly re-numbered in the text. This has now been updated in the original article.

The publishers apologise for any inconvenience caused by this error.

